# Successful Treatment of Rectovaginal Fistula Complicating Ulcerative Colitis With Infliximab: A Case Report and Review of the Literature

**DOI:** 10.14740/jocmr1987w

**Published:** 2014-10-16

**Authors:** Takako Nirei, Shinsuke Kazama, Masaya Hiyoshi, Nelson Hirokazu Tsuno, Takeshi Nishikawa, Toshiaki Tanaka, Junichiro Tanaka, Tomomichi Kiyomatsu, Keisuke Hata, Kazushige Kawai, Hiroaki Nozawa, Takamitsu Kanazawa, Hironori Yamaguchi, Soichiro Ishihara, Eiji Sunami, Joji Kitayama, Toshiaki Watanabe

**Affiliations:** a Division of Surgical Oncology, Department of Surgery, Faculty of Medicine, The University of Tokyo, 7-3-1 Hongo, Bunkyo-ku, Tokyo 113-8655, Japan; b Department of Blood transfusion, The University of Tokyo, 7-3-1 Hongo, Bunkyo-ku, Tokyo 113-8655, Japan

**Keywords:** Ulcerative colitis, Rectovaginal fistula, Anti-human tumor necrosis factor alpha antibody

## Abstract

Rectovaginal fistula is a rare complication of ulcerative colitis (UC) regardless of surgical history of rectum. Various surgical treatment modalities for the closure of rectovaginal fistula have been developed, but a radically curative therapy remains to be developed. Recently, infliximab, the chimeric anti-human tumor necrosis factor alpha (TNF-α) antibody, has been largely applied for the treatment of inflammatory bowel disease (IBD), and a few reports have shown its partial effectiveness in the management of rectovaginal fistulas associated with UC. In the present report, we describe the successful management of a rectovaginal fistula, following the stapled ileo-anal canal anastomosis in a UC patient, by administration of infliximab. The patient was a 40-year-old female, initially diagnosed as UC (total colitis type) at the age of 15. She received a restorative proctocolectomy at the age of 22, and developed a rectovaginal fistula at the eighth postoperative day. The surgical treatment of the fistula was repeated four times during the 10-year period, but it recurred in intervals ranging between 2 months and 5 years after the operation. The last recurrence occurred at the age of 32, but the surgical repair was considered difficult and a conservative management was indicated. At the age of 40, infusions of infliximab were started. Four weeks after the first infusion, drainage from the fistula was evidently reduced, and 2 weeks later, the fistula was completely closed. Thereafter, no recurrence of the fistula is observed, as confirmed by the abdominal magnetic resonance imaging (MRI) and the barium-enema study. From the present case, we concluded that infliximab may be an effective strategy for the management of fistulas associated with UC.

## Introduction

Rectovaginal fistula is usually associated with obstetric injury, crypto-glandular abscess, trauma, radiotherapy, operative complication, or inflammatory bowel disease (IBD). In IBD patients, it is more frequently observed in patients with Crohn’s disease (CD) than ulcerative colitis (UC). The incident rate of rectovaginal fistula complicating UC has been reported to be 0.5-2.2% [1, 2]. The treatment of rectovaginal fistula associated with IBD has been reported to be difficult. Therefore, it often requires repeated surgical procedures to achieve complete cure, or most of the cases will finally receive an ileostomy or proctocolectomy [1].

In the present case report, we demonstrate the case of a UC patient, in whom the rectovaginal fistula could be successfully managed by the administration of infliximab.

## Case Report

The patient was a 40-year-old female, initially diagnosed as UC (total colitis type) at the age of 15. She received a restorative proctocolectomy with the stapled ileo-anal canal J pouch anastomosis at the age of 22, but developed a rectovaginal fistula originating from the residual rectum at the eighth postoperative day. The surgical treatment of the fistula was repeated four times in our service (first was simple sutures and temporally ileostomy, second was transanal repair using mucosal flap, third was transanal repair using mucosal flap and temporally ileostomy, and fourth was transanal repair using mucosal flap) during the 10-year period, but it recurred in intervals ranging between 2 months and 5 years after the operation. The last recurrence occurred at the age of 32, but the surgical repair was considered to be difficult and a conservative treatment was indicated. At the age of 40, she developed frequent diarrhea (30 times/day), including bloody excrement, with consequent increasing of drainage from the rectovaginal fistula. Due to body weight loss associated with difficulty of dietary ingestion, she was admitted for a treatment.

**Figure 1:**
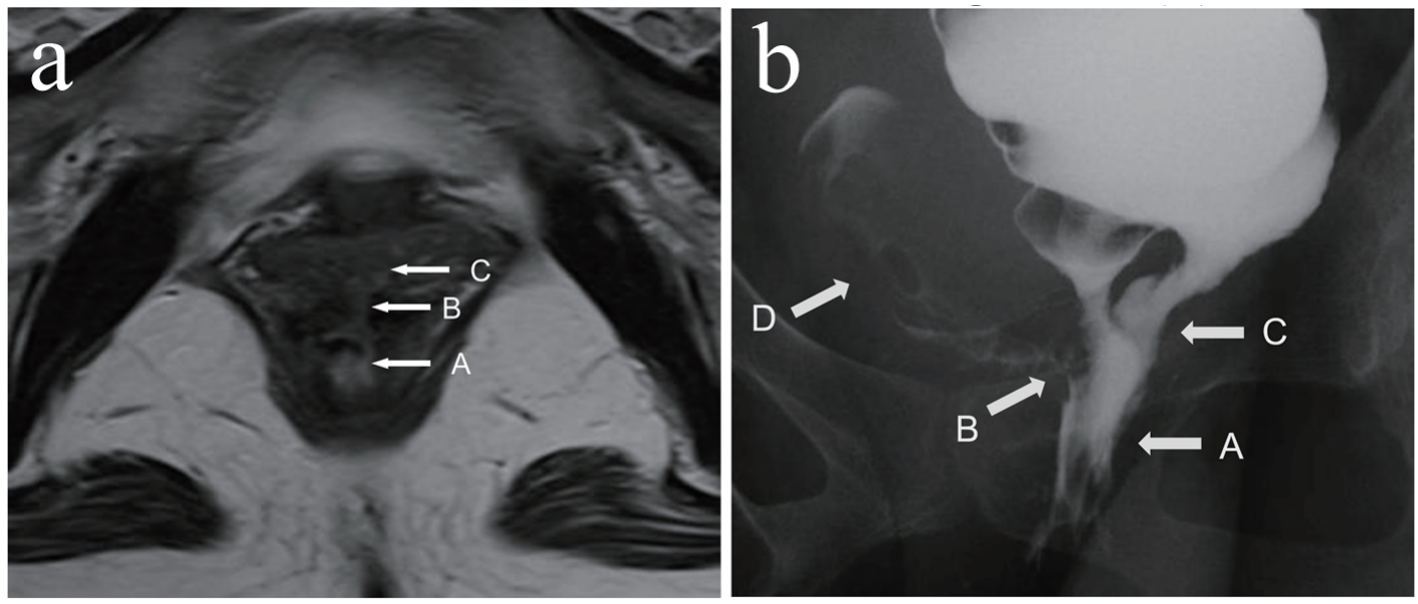
(a) MRI showed the rectovaginal fistula before administration of infliximab. (b) Barium-enema study showed the barium running into the vaginal cavity before administration of infliximab. White arrows pointed respectively A: rectum; B: rectovaginal fistula; C: J-pouch (atypical bridge); D: vagina cavity.

**Figure 2:**
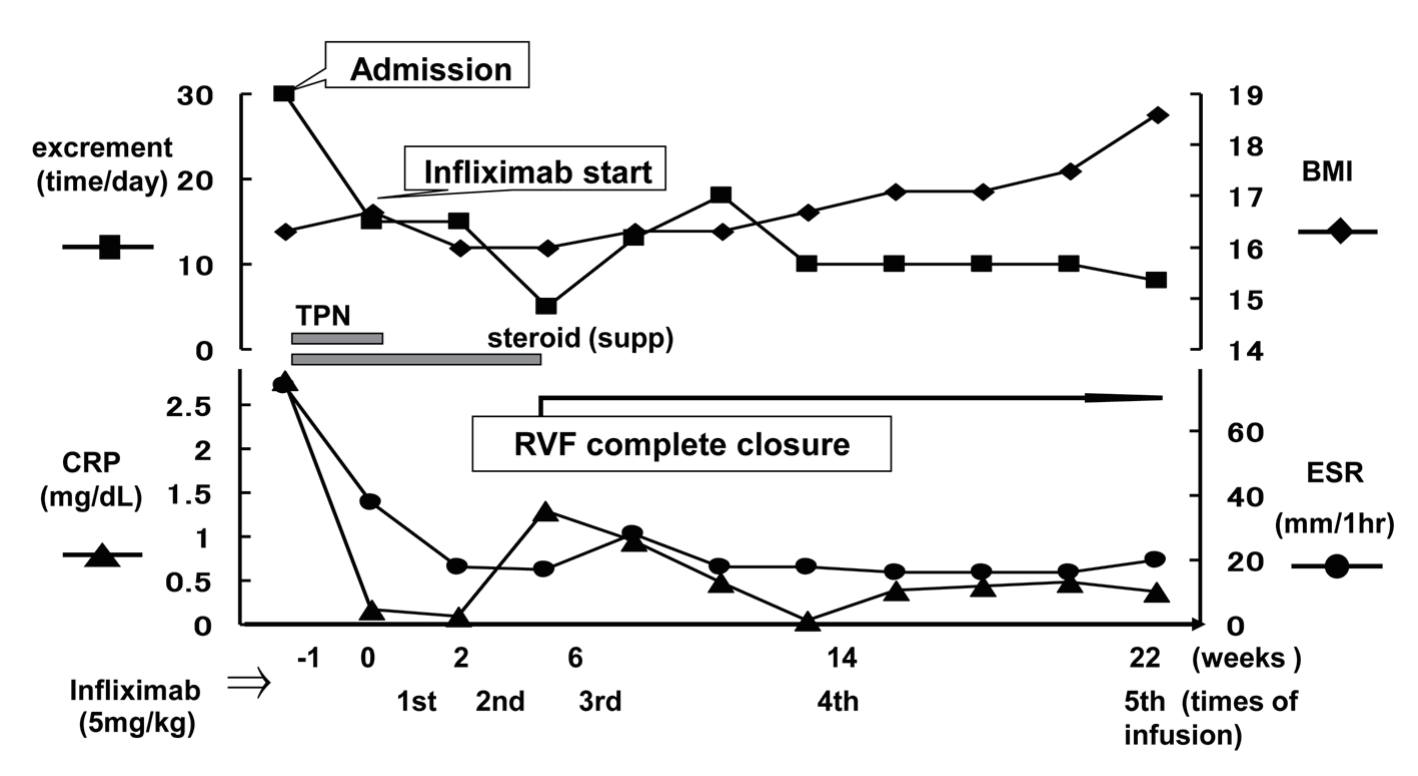
Symptomatic changes (excrement time, CRP, BMI, ERS) after admission in a graph with time dependently as a basic date (day 0) by the infliximab beginning day.

**Figure 3:**
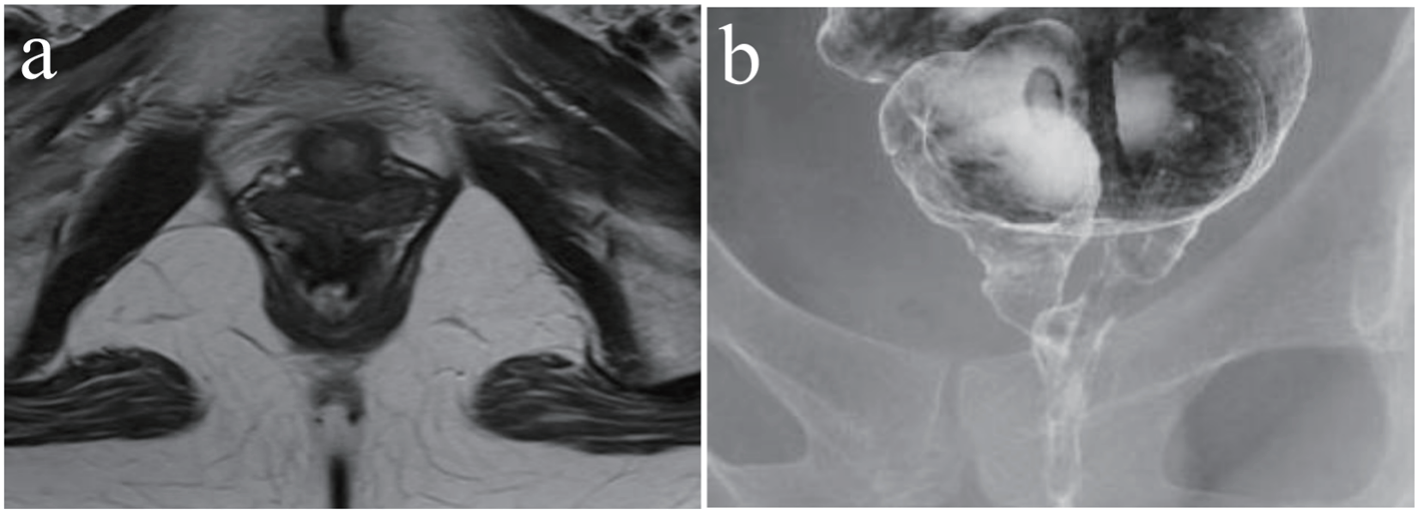
(a) MRI. (b) Barium-enema study. Both examinations are inspected from the start of therapy 1 year later, and confirmed rectovaginal fistula closing.

After the hospital admission, intravenous hyperalimentation with restriction of oral administration has been continued for 1 week. Though the dehydration and denutrition were significantly improved as well as the stool frequency, the symptoms from the rectovaginal fistula persisted. Figure 1 shows the abdominal magnetic resonance imaging (MRI) and barium-enema study of the rectovaginal fistula before starting treatment. Surgical treatment was considered unfeasible, therefore the conservative management with infusions of infliximab was advocated. After confirmed to be negative for tuberculosis and the informed consent was obtained, the infliximab-based treatment, consisting of intravenous infusions of infliximab at a dose of 5 mg/kg, at day 0 and weeks 2, 6, 10 and then every 8 weeks, was started. Figure 2 shows the time course of the symptomatic changes after the admission. Four weeks after the first infusion, drainage from the fistula was evidently reduced, and 2 weeks later, the fistula was completely closed. Thereafter, no recurrence of the fistula is observed for at least 26 months. We continue the administration of infliximab at the outpatient. Figure 3 shows the results of the imaging study 1 year after the start of infliximab therapy. The closure of the fistula was confirmed by the abdominal MRI and the barium-enema study.

## Discussion

Various complications are reported for UC, but it is deferent from CD, according to the previous reports, the frequency of rectovaginal fistula associated with UC was lower than that with CD [1-3]. Various techniques for the treatment of rectovaginal fistulas, including the loose draining seton, direct surgical repair, fistulotomy, fibrin glue instillation, endorectal or vaginal advancement flap, abdominal procedures with colorectal or coloanal anastomosis, and epiplooplasty, have been applied, according to the hole size of the fistula, their position, the sphincteric function, and their etiology [4, 5]. Since IBD patients with rectovaginal fistula have repeated inflammation of the rectum, the success rate of local surgical treatment has been reported to be very low [6]. Therefore, one of the most popular surgical procedures for the local control of rectovaginal fistulas in CD, namely endorectal advancement flap, has ultimately resulted in failure [7].

Recently, the monotherapy of infliximab, the chimeric anti-human tumor necrosis factor alpha (TNF-α) antibody, has been demonstrated to partially improve the rectovaginal fistulas associated with CD [8]. To the best of our knowledge, there are only two reports suggesting the effectiveness of infliximab for the treatment of rectovaginal fistulas developing after ileal pouch-anal anastomosis (IPAA) in UC [8, 9]. In the first report, infliximab (5 mg/kg, given at time 0 and weeks 2 and 6) with daily azathioprine (2.5 mg/kg) was administered in three UC patients, who had refractory pouchitis and rectovaginal fistula after the IPAA. In all cases, the fistulas had completely closed at 10 weeks after the start of infliximab [9]. In the second report, infliximab and/or immunomodulator (azathioprine/6-mercaptopurine) were administered to 13 UC patients, among the 382 who had received IPAA, complicated with various types of fistulas, including pouch-vaginal fistulas. The success rate was 66.7% (2/3) in those receiving infliximab only, 100% (1/1) in the case receiving immunomodulator only (1/1), and 44.4% (4/9) in those receiving both infliximab and immunomodulator, respectively. Therefore, from this report, the success rate of rectovaginal fistula treatment with infliximab was 54% (7/13) [10].

Although the exact mechanism of action of infliximab in controlling rectovaginal fistula still remains to be elucidated, it has been suggested to control the imbalance between pro-inflammatory and anti-inflammatory cytokines which is observed in the inflammatory pouch mucosa [8, 11]. Therefore, the control of the inflammatory state of the pouch mucosa by infliximab may have indirectly resulted in the closure of the fistula.

In our present case, the fistula was developed in rectum about 5 mm anal side from anastomosis, and the inflammatory of pouch mucosa was not observed. We consider that the fistula occurred and recurred due to active and chronic inflammatory in remnant rectum based on UC. The fistula was completely closed 6 weeks after the first infusion. Therefore, we are confident that infliximab importantly contributed to the closure of the rectovaginal fistula, possibly by controlling the inflammation of the remnant rectum. In this patient, the surgical control of the fistula was thought to be unfeasible, and infliximab administration significantly improved her quality of life.

In conclusion, various surgical procedures have been developed to repair rectovaginal fistula associated with UC; however, there is a lot of recurrent case. From our case, infliximab may be an effective strategy for the management of fistulas associated with UC.
